# Rheological Properties of Graphene Modified Asphalt Binders

**DOI:** 10.3390/nano10112197

**Published:** 2020-11-04

**Authors:** Lu Yang, Dunhong Zhou, Yang Kang

**Affiliations:** 1 College of Water Resources and Architectural Engineering, Northwest A&F University, Yangling 712100, China; 184@nwafu.edu.cn; 2 Nanjing ASFT Neomaterials LLC, Xinmofan Road, Nanjing 210009, China; dhzhou1990@foxmail.com

**Keywords:** graphene, modified asphalt, percolation, rotational viscosity, rheological measurements

## Abstract

Recently, low-cost, high-quality graphene can be obtained readily, so it is potential to prepare conductive graphene modified asphalts (GMAs). In this paper, GMAs were prepared with 0%, 2%, 4%, 6%, 8%, and 10% of graphene by weight of composites. Dynamic shear rheological experiments conducted from −30 to 120 °C illustrate that elasticity at above ambient temperatures and rutting resistance at higher temperatures are enhanced and, especially, the conceived percolation of GMAs occurs at graphene contents (GC) above 8% which were verified from three changes as GC increases, i.e., the curve characteristics of complex moduli, storage moduli at temperatures over 100 °C, temperatures when the phase angle reaches 90° and the trend of *T*_G′=G″_. The modification mechanisms are different before and after percolation. Before the percolation threshold, graphene which has a molecular structure similar to asphaltene enhances asphalt, like increasing asphaltene components, and after threshold, graphene improves asphalt because of the formed graphene networks. Rotational viscosities test results show that the higher the GC is, the higher the operating temperatures are, but the operating temperatures are higher than 200 °C when GC is above 4%. The percolation helps to further develop conductive asphalt concrete for intelligence pavement, but the operating properties of GMAs need to be improved.

## 1. Introduction

Graphene is a kind of magic nano-material. No matter what material it is added to, it seems to have a positive impact on the properties of the material [1,2,3,4,5,6,7,8,9,10,11]. Graphene has excellent mechanical properties [12]. Moreover, the structures of graphene and asphaltene are similar, and graphene has an enormous specific surface area and an intense physical adsorption effect with asphalt, so they have good affinity [13,14]. Nonetheless, the applications of graphene in the field of road materials are rare, but graphene is fast becoming a key potential modifier in achieving more durable and intelligent asphalt materials [15,16,17].

Moreno-Navarro, F. et al. [15], Yang, Q.L. et al. [17], Nazki, M.A. et al. [18] and Hafeez, M. et al. [19] carried out rheological experiments on a variety of control asphalts and GMAs with graphene content less than 4% (determined by weight). It was demonstrated that compared with base asphalt, there is an increase in the complex shear modulus and rutting resistance and a reduction in phase angle. With no accident, Hafeez, M. et al. [19] also found that the modification effect of 4% graphene nanoplatelets (GNPs) is more obvious than that of 2% GNPs. Le, J.L. et al. [20] conducted electrical conductivity on asphalts reinforced with 0%, 1.5%, 3%, 6%, 10% by the weight of the binders, and they supposed that the inhomogeneous dispersion of GNPs in binders led the failure of conductivity, although the amount of GNP addition is beyond the theoretical percolation threshold.

It is noteworthy that the modification effect of low-content GMAs is not as good as that of polymers. According to the percolation theory, when the concentration of graphene exceeds a certain value, the GMAs’ system performance will take place with an abrupt change. Perhaps, graphene will present its magic properties when it is added with a higher content. However, previous studies of GMAs have not dealt with the performances of higher-content GMAs. The reason for this may be that the price of graphene was too expensive in the near past. To our excitement, Luong, D.X. et al. obtained high-quality and low-defect graphene through fast Joule heating [21], which is a scalable method to prepare very low-cost, high-quality graphene [15,22]. Little conclusive information has been provided with regard to the rotational viscosities of GMAs, which greatly impact engineering application. The temperature range of the mechanical properties involved is relatively narrow.

This paper aims to characterize the application potential of high-content GMAs in a wider temperature range, including mechanical properties, working performances, and the relationship between the microstructure of GMAs and the graphene content. For this purpose, strain sweeps, temperature sweeps and the rotational viscosity tests were conducted by dynamic shear rheometer (DSR) to comprehensively evaluate the different graphene content of GMAs. By analyzing the above results, the influence of graphene on the viscoelasticity, the mixing and compaction temperature of GMAs were determined, based on which the application potential of GMAs in engineering was evaluated, which laid a solid foundation for the future intelligent pavement application of GMAs.

## 2. Materials and Methods

### 2.1. Materials

**Figure 1:**
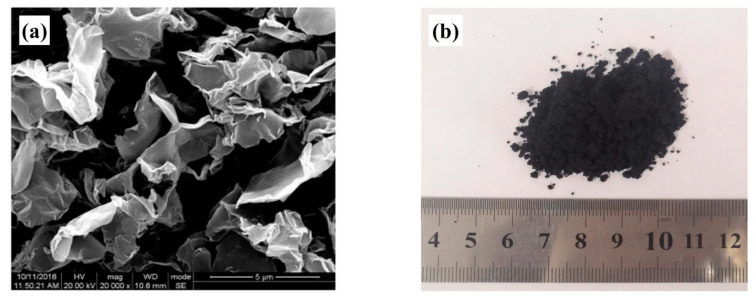
(**a**) SEM of Industrial Reduced Graphene Oxide (source: the official website of Chengdu organic chemicals Co. Ltd. Chinese Academy of Sciences). (**b**) A picture of graphene powder.

Graphene was purchased from Zhongke Time Nano (Chengdu, China), which is in powder form. The relevant parameters are listed in Table 1, the scanning electron microscope (SEM) (Hitachi, Japan) results are shown in Figure 1a, and the picture of graphene powder is shown in Figure 1b. The 70# asphalt was selected as control asphalt. Properties are listed in Table 2.

### 2.2. Preparation of GMAs

Firstly, the calculated and weighed control asphalt (total of 70 g of graphene and asphalt) was put into the oven at 120 °C for preheating. After that, it was quickly transferred to a constant temperature silicon oil bath at 145 °C for heating to make it reach the melting state. Then, the corresponding proportion of graphene was slowly added into the molten control asphalt in batches, while the graphene and asphalt were stirred at low speed. For 0%, 2% and 4% content of GMAs, they were stirred at 300 rpm for 20 min, 500 rpm for 20 min, 1000 rpm for 20 min, 2000 rpm for 20 min, and 2500 rpm for 40 min in a silicone oil bath at 145 °C to ensure it was homogeneous. For 6%, 8% and 10% content of GMAs, it was kept at constant temperature in a 150 °C silicone oil bath (because the viscosity of modified asphalt is too large to be stirred at low temperatures), the stirring process was the same as above, and, finally they were kept at high temperature until the bubbles disappeared, and then cooled to room temperature.

### 2.3. Test Methods

#### 2.3.1. Strain Sweeps

The linear viscoelastic regions (LVEs) of GMAs were determined by using DSR, MCR 302 (Anton Parr Inc., Graz, Austria). For asphalt, the higher the temperature is, the longer the LVE plateau is [23]. In view of the temperature sweep range (−30–120 °C), this paper selected −30 °C to carry out a strain sweep. The diameter of the parallel plate is 8 mm (PP08) with the gap of 1 mm, oscillation frequency of 10 rad/s [24], and strain increase from 0.001 to 10%. Checking time continued for 5 min to eliminate thermal history and attain an equilibrium state at each measurement [25].

#### 2.3.2. Temperature Sweeps

In order to explore the mechanical properties of asphalt at high and low temperatures, the temperature sweeps of GMAs were carried out by DSR, MCR 302(Anton Parr Inc., Graz, Austria). Temperature sweeps were performed with strain of 0.01%, oscillation frequency of 10 rad/s, and the temperature range reduced at the interval of 1 °C/min from −30 to 120 °C. A 25 mm parallel plate (PP25) was used to determine the overall trend of GMAs in the temperature range of −30–120 °C. In order to further analyze the results, in the temperature range from −30 to 35 °C, an 8 mm parallel plate with 1 mm gap was used, and when the temperature range was from 35–120 °C, a 25 mm parallel plate with 1 mm gap was used. Checking time continued for 10 min to eliminate thermal history and attain an equilibrium state at each measurement.

#### 2.3.3. Rotational Viscosity Tests

**Figure 2:**
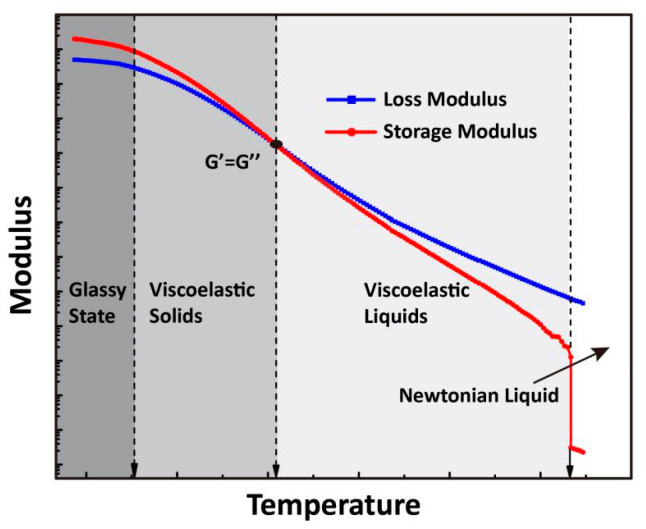
Four processes of asphalt binder mechanical state changing with temperature (the loss moduli approach the maximum plateau over the whole range of temperatures, the corresponding temperature is the glass transition temperature *T*_g_; at temperature when G′ = G″, viscoelastic asphalt transforms from viscoelastic solid to viscoelastic liquid; with G′ of asphalt decreases rapidly, asphalt changes from viscoelastic liquid to pure viscous Newtonian fluid) [23].

In order to determine the suitable construction temperature of asphalts and explore the engineering feasibility of GMAs, the rotational viscosities of different content of GMAs were measured by MCR 302. As is known to all, asphalt is a kind of viscoelastic material. There is a glass-forming process of Newtonian fluid state to glassy state, which is shown in Figure 2. According to the results of temperature sweeps, the transition temperature from viscoelastic liquid to Newtonian fluid is distinctly different for control asphalt and different-content GMAs. In order to improve efficiency and accurately measure the viscosities of GMAs, the temperature test ranges in this paper are diverse. A 25 mm parallel plate with a 1 mm gap was utilized. The rotation frequency is 5.2 rpm, with checking time continued for 10 min to eliminate thermal history and attain an equilibrium state at each measurement.

### 2.4. Percolation Model

**Figure 3:**
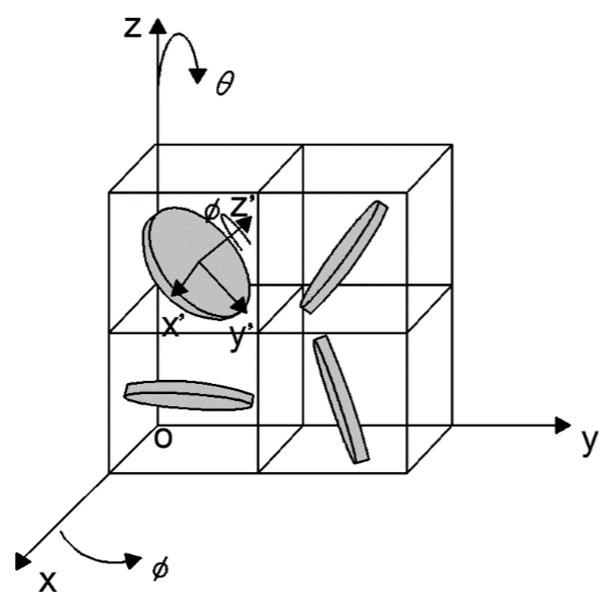
Schematic drawing of the IPD model for 3D distribution of fillers with high aspect ratios [26].

Li and Kim [26] proposed the IPD (based on the average interparticle distance) model to predict the percolation threshold of conducting polymer composites containing disc-shaped nanoparticles with high aspect ratios. Figure 3 is the IPD model for 3D distribution of fillers with high aspect ratios, in which a typical individual particle is modeled as a thin and round platelet, and then the composite is divided into cubic elements, each containing one particle in the center, where the total number of cubic elements is equal to the total number of particles. Three Eulerian angles (*θ*, *Φ*, *Ψ*) describe the random orientation of the platelets. Thus, the percolation threshold of nanocomposites, containing 3D, random-distributed, disc-shaped nanoparticles based on the average interparticle distance approach, can be calculated by the following formula

(1) $$V_{tr}=\frac{27\pi d^{2}t}{4{\left(d+S_{ip}\right)}^{3}}$$

where *V_tr_* is the threshold of volume fraction of particle, *d* is the diameter of the graphene disk, *t* is the thickness of the packed graphene disk, and *S_ip_* is the maximum spacing that allows electron-hopping to take place between adjacent conductive fillers owing to quantum-mechanical tunneling. The *S_ip_* is expected to be in the order of 10 nm. For the graphene and asphalt binder used in this paper, (please see the specific data in Table 1 and Table 2) according to the formula, *V_tr_* ≈ 3.5%, and the corresponding to mass ratio is about 7.6%. Of course, this is only a theoretical estimate. It is worth noting that the current cubic-element model with homogeneously distributed platelets can only be applied to a system containing a filler content below the percolation threshold.

When the content of filler exceeds the percolation threshold, there have been marked changes in some measurable properties of the investigated system. There are many models describing this percolation. Kirkpatrick-Zalle [27] utilized the gel theory of Flory to describe the formation of conductive network, and put forward the classical statistical percolation theory equation near the percolation threshold

(2) $$\sigma =\sigma_{p}{\left(\upsilon -\upsilon_{c}\right)}^{X}$$

where σ is the electrical conductivity of composite, $\sigma_{p}$ is electrical conductivity of the fillers, ν is volume fraction of filler, $\upsilon_{c}$ is percolation critical volume fraction, and X is a coefficient related to system dimensions. This paper presents a percolation model of temperature related to the rheological properties of GMAs, versus the mass fraction of graphene fillers, which is very similar to this classical statistical percolation equation.

### 2.5. Arrhenius Equation

According to the fragile liquid theory, asphalt binders are of typical fragile liquids. Thus, their temperature dependences of viscosity satisfy the Vogel–Fulcher–Tammann (VFT) equation

(3) $$\eta =\eta_{0}e^{\left(\frac{DT_{0}}{T-T_{0}}\right)}$$

where *η* is the viscosity and *D*, *T*_0_ and *η*_0_ are the constants of the VFT equation [23]. When in a certain temperature range where asphalt binders experience different mechanical states, e.g., viscous liquid and viscoelastic liquid, it is hard to find a solid linear extrapolation relationship. Fortunately, when temperature range is not across the transition temperature between the viscoelastic state and the viscous state, such as where asphalt is only in the Newtonian state or only in the viscoelastic state, the dependence between the asphalt viscosity and temperature follows the Arrhenius equation [28]

(4) $$\eta =Ae^{\frac{-Ea}{RT}}$$

where *η* is the viscosity at *T* (Pa·s). *A* is a regression constant, *E_a_* is the visco-flow activation energy (kJ·mol^−1^), *R* is the universal gas constant (8.314 J·mol^−1^·K^−1^), *T* is temperature in degrees K.

Take the logarithm from both sides of Equation (4) that is transformed into a linear relationship between the logarithmic value of the viscosity lg (*η*) and the reciprocal of the temperature 1/*T*, we can obtain

(5) $$\mathrm{lg}\eta =\mathrm{lg}A-\frac{0.43E_{a}}{RT}$$

where the slope of the straight line is 1/*T* coefficient value, that is, $-\frac{0.43E_{a}}{R}$, and then *E_a_* can be acquired, which can characterize the thermal susceptibility of asphalt in the corresponding temperature ranges.

## 3. Results and Discussion

### 3.1. Rheological Analysis

**Figure 4:**
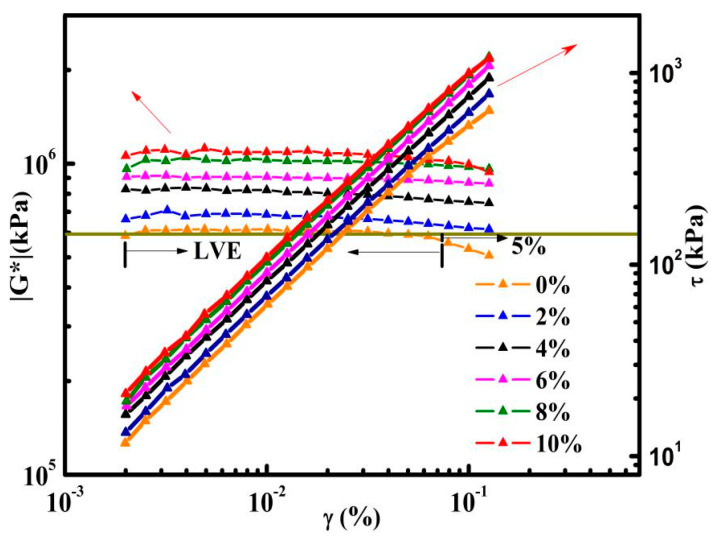
Strain sweep results of control asphalt and graphene modified asphalts (GMAs) at −30 °C.

As shown in Figure 4, the LVEs of asphalts were determined by strain sweep. The figure leads us to the conclusion that when the complex shear modulus (|G*|) deviates from its average value within 5% [29], |G*| of control asphalt and GMAs are in the platform area, that is, all samples are in the LVEs range when strains are less than 0.05%. In consideration of the above, 0.01% was chosen as the strain of temperature sweeps.

**Figure 5:**
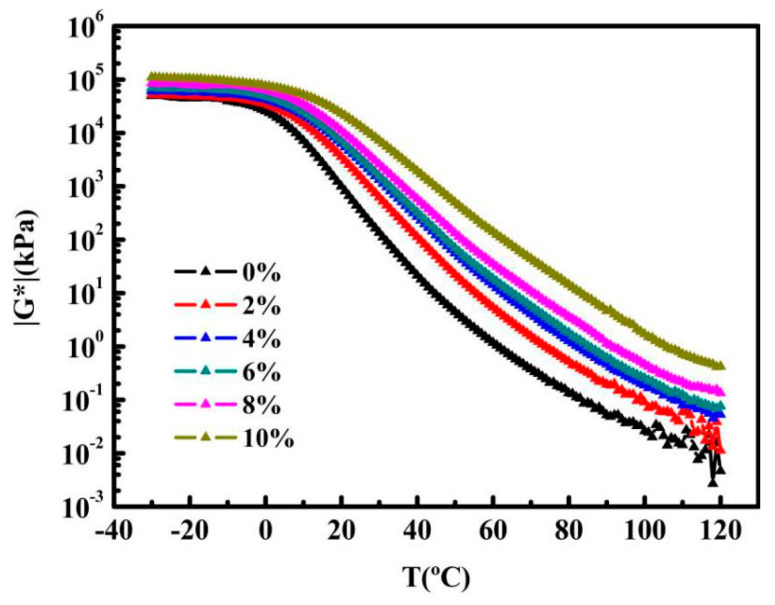
Temperature sweeps results of control asphalt and GMAs (complex modulus versus temperature) at −30–120 °C (PP25).

It can be seen from Figure 5 that |G*| of control asphalt and any content of GMAs declines with the increase of temperature from −30 to 120 °C. On account of the rising temperature, the degree of interaction between each other weakens, with the molecules in the substance moving violently, thus the resistance to deformation declines. |G*| is closely linked to the content of graphene. The higher the content of graphene is, the higher the complex modulus is, which is an evidence of the presence and superiority of the graphene in the GMAs. The graph proves the dominance of GMAs, compared with the control asphalt.

It is worthy of notice that at graphene content above 8%, |G*| of GMAs at above 100 °C remain stable, which differ from those of graphene content below 8%. This implies that the percolation of GMAs system occurs and graphene networks form at graphene content above 8%, and also, 8% is very close to the theoretical value.

**Figure 6:**
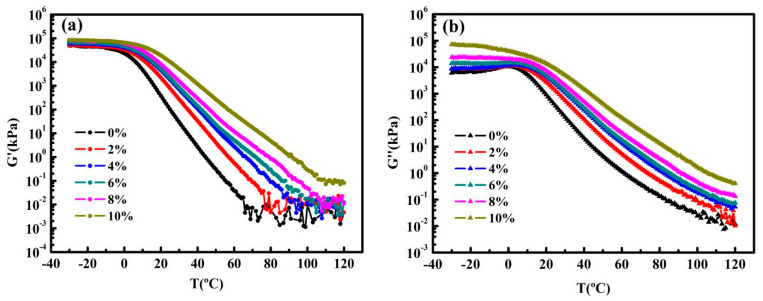
(**a**) Temperature sweeps results of control asphalt and GMAs (storage moduli versus temperature) at −30–120 °C (PP25). (**b**) Temperature sweeps results of control asphalt and GMAs (loss moduli versus temperature) at −30–120 °C (PP25).

Figure 6a,b precisely provide some data regarding the storage (G′) and loss moduli (G″) with the content of graphene. As can be seen from the diagram, the G′ and G″ of asphalts simultaneously increase with the content of graphene, which is consistent with the trend of |G*|. It is worth mentioning that G′ of control asphalt and modified asphalts (2%, 4%, 6%, 8%) visibly fluctuate at high temperature, likewise the G″ fluctuates slightly (0%, 2%), which mean that the absolute values of G′ and G″ are too small to determine accurately, especially values of G′. The temperature which G′ begins to fluctuate is diverse (this temperature point corresponds to that the phase angle reaches 90°, as mentioned later). At the moment where they start to enter the Newtonian fluid little by little, the storage modulus is almost zero, and hence there is a large fluctuation. The reason for the undulation is that the content of graphene is too low, which is not as good as that of high-content GMAs, in which the continuous phase of the graphene network formed and the solid graphene networks provide the moduli at higher content.

To be honest, using the same geometry has disadvantages, as the complex modulus from −30–120 °C cannot be covered due to the limitation of the geometry itself. In order to confirm the experimental results, we use different geometries to carry out experiments in two stages, i.e., PP08 is used for −30–35 °C and PP25 is used for 35–120 °C. The results show that from −30 to 120 °C, the overall trend of complex modulus is consistent, and from 35 °C to 120 °C, the complex modulus curves are completely coincident, while from −30 to 35 °C, the complex modulus absolute values are different on account of the different thermal histories of asphalt samples.

**Figure 7:**
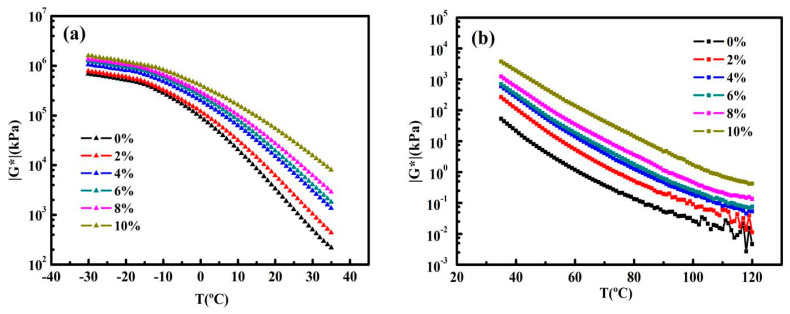
(**a**) Temperature sweeps results of control asphalt and GMAs (complex modulus versus temperature) at −30–35 °C (PP08). (**b**) Temperature sweeps results of control asphalt and GMAs (complex modulus versus temperature) at 35–120 °C (PP25).

Figure 7a,b show that the effect of graphene on |G*| of asphalts at medium and high temperatures are more distinct than that at lower temperatures, which is consistent with the conclusions of other researchers [30,31]. In detail, compared to that of control asphalt, in the temperature section (−30–0 °C), |G*| of all samples is in the same order of magnitude, as to the temperature section (35–120 °C), |G*| increases by 1 to 3 orders of magnitude (even at 0–35 °C, this trend still exists). The tremendous difference reflects the thermal stability of the graphene molecular frame at high temperatures, especially at the higher graphene contents. Moreover, the denser the graphene sheets are, the more stable the mechanical properties of GMAs at high temperatures become, and it can be inferred that the graphene effect on the asphalts could be depicted as an enhancement of high-temperature rutting resistance.

**Figure 8:**
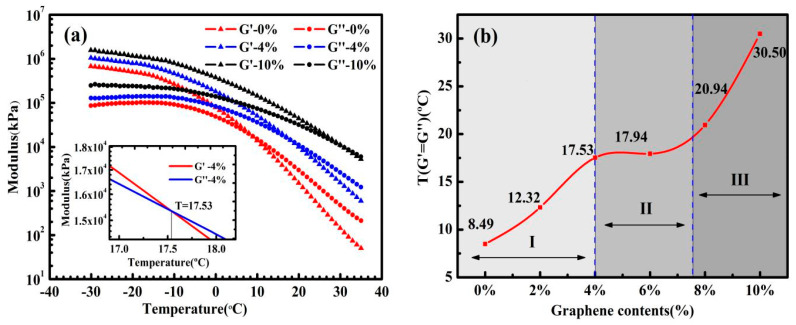
(**a**) Temperature sweep results of control asphalt and GMAs (modulus versus temperature) at −30–35 °C, the content of graphene is 0%, 4%, and 10%. (**b**) The temperature change of G′ = G″ of control asphalt and GMAs with graphene contents.

**Figure 9:**
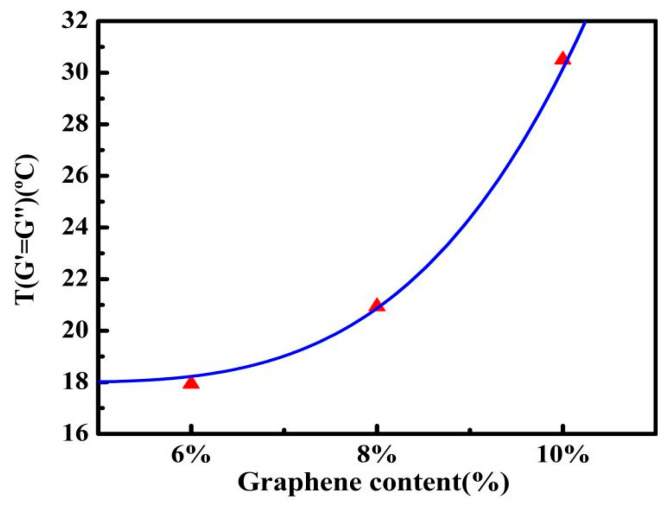
*T*_G′=G″_ versus graphene content after percolation threshold, and fitted curve by an equation similar to classical percolation theory equation.

As shown in Figure 8a, there is an intersection point between G′ and G″, that is, G′ = G″. As mentioned in the experimental method, this is the point at which asphalt transforms from viscoelastic solid to viscoelastic liquid. It is apparent that when the temperatures are lower than that at this point, G′ is dominant and, on the contrary, G″ is dominant. Especially, the tendency of this temperature (*T*_G′=G″_) with the increasing of graphene content is in concert with that of the glass transition temperature, and this temperature is much easier accurately verify. As shown in Figure 8b, the change in *T*_G′=G″_ with graphene content can be obviously divided into three domains. In domain I, graphene, which has a molecular structure similar to asphaltene, enhances the asphalt to some extent, like increasing asphaltene to the asphalt, thus, when graphene content is from 0% to 4%, *T*_G′=G″_ rises about 4 °C with every 2% graphene increase. In domain II, at the graphene content from 4–6%, there is a plateau of *T*_G′=G″_ at about 18 °C. This is a strong implication that there is the percolation threshold at graphene content about 8%, which is in accord with the inferred result from Figure 5 and the theoretical calculated value of 7.6%. In other words, at above about 8%, i.e., the percolation threshold, graphene phase within the asphalt becomes continuous, and the graphene network forms, which has the potential to prepare future conductive asphalt concretes. In domain III, there is a rapid increase in *T*_G′=G″_ in the same content step increasing, i.e., when graphene content increases from 8% to 10%, *T*_G′=G″_ improves about by 10 °C, that is, GMAs enter another phase, in which graphene structure plays a crucial role. Similar to the classical percolation theory equation introduced in Section 2.4, *T*_G′=G″_ versus graphene content after percolation threshold are fitted, as shown in Figure 9, by an equation as follows

(6) $$T=18+0.3*{\left(G-0.076\right)}^{3.45}$$

where *T* is *T*_G′=G″_, 18 is the base value of transition temperature *T*_G′=G″_ as shown in Figure 8b, 0.3 is a characteristic constant related to the mechanical properties of GMAs, G is content of graphene by weight, 0.076 is the calculated theoretical penetration threshold, 3.45 is coefficient related to system dimensions, which means the interlinked percolation network of graphene inside the GMAs system is above three dimensions. We speculate that when the content of graphene exceeds 10%, for example, 12%, the *T*_G′=G″_ will be higher than 50 °C. However, due to the viscosity of GMA with high-content graphene is too large to stir homogenously, thus, the content of graphene in this study is limited to 10%.

**Figure 10:**
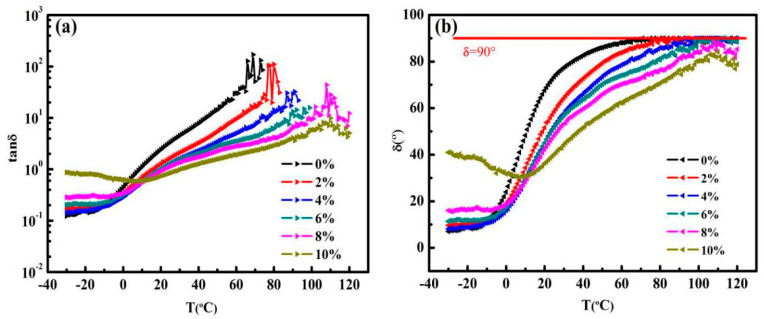
(**a**) Damping factor versus temperature from −30 to 120 °C. (**b**) Phase angle versus temperature from −30 to 120 °C.

Figure 10a shows that damping factor (tanδ) gradually increases with the rise in temperature for both control asphalt and GMAs, which reveals that under the action of loading, the viscous component of asphalt increases, i.e., the irreversible component in deformation increases. This will lead to the asphalt being prone to permanent deformation. What is surprising is that tan δ shows a decreasing trend when the graphene content is on the rise, which results in the elastic response of asphalt improving and the viscosity response reducing, thereby leading to the enhancement of asphalts’ deformation resistance and improving rutting resistance. Figure 10b unfolds a clear comparison between control asphalt and GMAs with regard to phase angle δ. Upon δ reaching 90°, the elastic properties of control asphalt and GMAs are basically lost. The fluctuation can clearly be ascribed to the above phenomena. The temperature at the phase angle reaching 90° raises by degrees with the rise in the content of graphene. The temperature corresponding to the δ reaching 90° of 0%, 2%, 4%, 6% content of GMAs is 69, 77, 92, 100 °C respectively, nevertheless 8%, 10% content does not even reach 90° in the experimental temperature, implying that the GMAs with high graphene content maintain viscoelasticity in a large temperature range and have good high temperature stability. It is noteworthy that this is new evidence that with graphene content at about 8%, there is a percolation threshold.

### 3.2. Rotational Viscosity Tests

**Figure 11:**
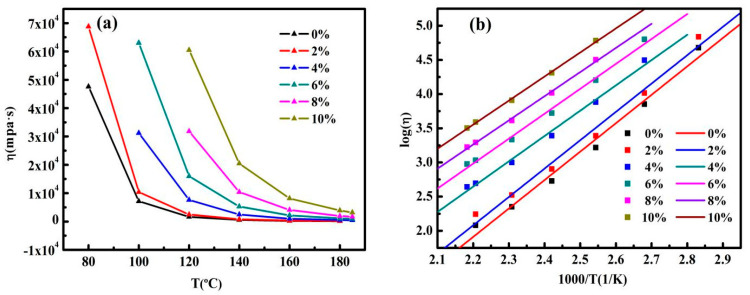
(**a**) Temperature dependence of viscosity of control asphalt and GMAs. (**b**) log (*η*) versus reciprocal temperature for control asphalt and GMAs.

The viscosity of an asphalt binder at high temperature is considered to be a significant characteristic to affirm the working temperature. For the sake of ensuring that the asphalt has sufficient mobility in the process of pumping and mixing, the mixing and compaction temperature were determined by measuring the rotational viscosities of control asphalt and GMAs [32,33,34]. According to Figure 11a, not surprisingly, the viscosity (*η*) of all asphalt falls with the increase in temperature. Besides, at the same temperature, in comparison to the control asphalt, the viscosity of GMAs comes up along with the increase in graphene content. However, the viscosity of all asphalts is close at 180 °C, which may be due to the weakening of the influence of nanoparticles on the viscosity at higher temperature.

**Figure 12:**
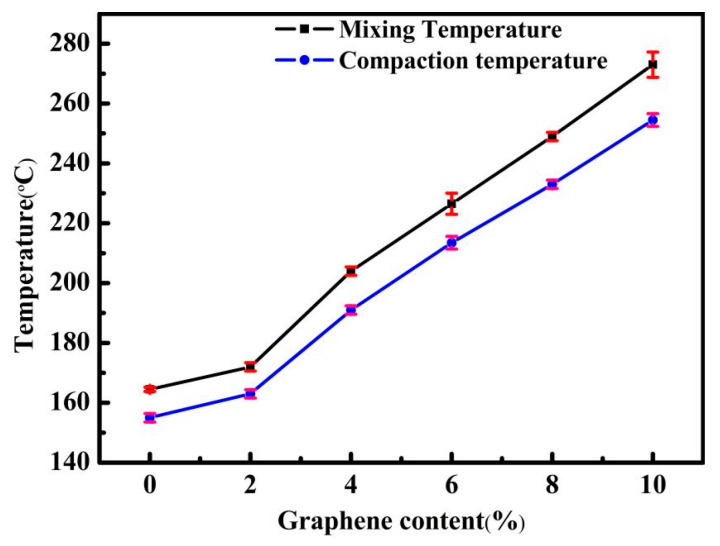
Mixing temperature and compaction temperature of control asphalt and GMAs.

From the construction point of view, the most suitable viscosity range for asphalt mixing and compaction is (0.17 ± 0.02) Pa·s, (0.28 ± 0.03) Pa·s [35]. In accordance with the temperature when the phase angle δ reaches 90°, all asphalt entered Newtonian fluid within the viscosity test range. The viscosity–temperature curves can be fitted by the Arrhenius equation, and for control asphalt and GMAs, the specific values of relevant parameters in the formula and R^2^ are shown in Table 3. It is easy to see from Table 3 that the activation energy decreases with the increase in graphene content, indicating that it is not vulnerable to temperature changes in the testing temperature range for high-content GMAs, i.e., improving the thermal susceptibility and deformation resistance after adding graphene at higher temperatures [36]. The relationship between the logarithmic value of the viscosities of all asphalts and the reciprocal of temperature is shown in Figure 11b. The mixing and compaction temperatures of control asphalt and GMAs can be deduced from Equation (5), and the results are listed in Figure 12. The addition of graphene enhanced the mixing and compaction temperature of asphalts, which was embodied in that the higher the content of graphene is, the higher the viscosity of asphalt is. Unfortunately, asphalts with high viscosity will give rise to excessive energy consumption and asphalt aging in the construction process.

## 4. Conclusions

To evaluate the potential for the intelligent pavement and modification effect of high-content graphene on asphalts, rheological experiments and rotational viscosity tests were carried out on the control asphalt and GMAs with 2%, 4%, 6%, 8% and 10% of graphene by weight. On this basis, we conclude that:

(1) At graphene content above 8%, the |G*| of GMAs at above 100 °C remains stable, which differs from those of graphene content below 8%. This implies that the percolation of GMAs occurs and graphene networks form at graphene content above 8%, which means that it is possible to make conductive asphalt concrete when the graphene content is higher than 8%;
(2) The GMAs’ modification mechanisms are different before and after the percolation point, i.e., before the percolation threshold, graphene, which has a molecular structure similar to asphaltene, enhances asphalt to some extent, like increasing asphaltene, and after the percolation threshold, graphene improves asphalt because of the formed graphene networks;
(3) In the temperature section of −30–120 °C, in comparison to control asphalt, as the graphene content increases, the moduli of GMAs rises gradually, and the phase angle and damping factor decrease. This illustrates that graphene boosts the elasticity and the high-temperature rutting resistance of asphalt;
(4) The differences of moduli at higher temperatures between the GMAs and the control asphalt are greater than those at lower temperatures, predicting that the modification effect of graphene at higher temperatures is superior than those at lower temperatures, which is because the temperature susceptibility of graphene is much lower than that of control asphalt, and the rigid molecular structure of graphene frames GMAs at higher temperatures;
(5) The temperature at δ reaching 90° rises gradually as a result of the increase in graphene content, indicating that the temperature of asphalt from viscoelastic fluid to Newtonian fluid is on the increase. When GMAs’ contents are 8% and 10%, they do not even reach 90° in the experimental temperature range. From this perspective, graphene enhances the strength and the high-temperature rutting resistance. This is also evidence that there is a percolation threshold at graphene content above 8%;
(6) In terms of viscosity properties, it has been shown that adding graphene promotes the viscosity of all asphalt. The higher the content of graphene is, the bigger the viscosity is. The higher the temperature is, the closer the viscosity of different graphene content of GMAs is. Obviously, graphene improves the mixing temperature and compaction temperature of asphalts, which should be improved in the future to facilitate the pavement construction.

## Figures and Tables

**Table 1:** Physical properties of graphene.

Purity	Layers	Specific Surface Area	Scale	Electrical Conductivity
>97 wt %	<10	80–120 m^2^/g	<6 μm	>1000 S/m

**Table 2:** Physical properties of 70# asphalt.

Properties of Asphalt		Method
Density@15 °C/g cm^−3^	1.034	JTG E20-2011 T0603-2011
Penetration@25 °C/0.1 mm	68.0	JTG E20-2011 T0604-2011
Softening point (°C)	48.5	JTG E20-2011 T0606-2011
Viscosity@60 °C/Pa s	218.2	JTG E20-2011 T0625-2011
Ductility@15 °C/cm	>150	JTG E20-2011 T0624-2011
Flash point (°C)	328	JTG E20-2011 T0611-2011
Fraass breaking point (°C)	−17.5	JTG E20-2011 T0613-1993

**Table 3:** *A* and *E_a_* in Arrhenius equation for control asphalt and GMAs and R^2^.

Graphene Contents (%)	*A*	*E_a_* (kJ·mol^−1^)	R^2^
0%	5.75 × 10^−8^	80.43	0.982
2%	8.91 × 10^−8^	80.24	0.984
4%	3.16 × 10^−6^	71.54	0.991
6%	9.12 × 10^−6^	70.57	0.993
8%	3.09 × 10^−5^	68.25	0.997
10%	6.46 × 10^−5^	68.06	0.998
